# Decreased Response to Positive Facial Affect in a Depressed Cohort in the Dorsal Striatum During a Working Memory Task—A Preliminary fMRI Study

**DOI:** 10.3389/fpsyt.2019.00060

**Published:** 2019-03-05

**Authors:** Peter Goodin, Gemma Lamp, Matthew E. Hughes, Susan L. Rossell, Joseph Ciorciari

**Affiliations:** ^1^Centre for Mental Health, School of Health Sciences, Swinburne University, Hawthorn, VIC, Australia; ^2^Melbourne Brain Centre @ Royal Melbourne Hospital, Parkville, VIC, Australia; ^3^School of Psychology and Public Health, Latrobe University, Bundoora, VIC, Australia

**Keywords:** fMRI, working memory, emotional processing, dorsal striatum, depression, n-back

## Abstract

People with depression have shown alterations in processing emotional information and working memory functionality. There is some evidence that emotional content may interact with working memory update processes, however neurological correlates are current unknown. In this preliminary study we utilized a novel version of the emotional variant of the n-back working memory task in fMRI. We examined BOLD response of 14 healthy controls and 13 depressed participants in response to happy, sad, and neutral displays of facial affect. No accuracy or reaction time differences were found between the two groups. The depressed group showed significantly decreased BOLD response to happy faces compared to the control group areas of the dorsal striatum and anterior cingulate. Significant, moderate, positive associations were found between right caudate activation with anxiety score and anterior cingulate activation with depression score in those with depression. Our novel task was able to elicit group level differences in emotional processing during working memory update. These results suggest those with depression fail to differentiate between positive emotional stimuli and stimuli with no emotional content.

## Introduction

Depression is a common mood disorder that significantly impacts the lives of an estimated 16% of the global population (1, 2). While primarily associated with a host of physical and internal emotional states, alterations to cognition and processing of emotional information are also commonly reported (3–6). One area of impaired cognitive ability in depression that has received widespread examination is working memory (WM). WM is thought to be a limited capacity sensory register and temporarily holds incoming information for manipulation and more permanent storage in long term memory (LTM) (7). As new information is brought in, existing information is either passed to LTM or discarded, making successful updating an important part of general cognition. Multiple brain regions have been identified with normal WM functioning (8–13) including the medial cerebellum, bilateral medial parietal, bilateral premotor, bilateral dorsolateral, and ventrolateral prefrontal cortex (DLPFC & VLPFC, respectively) and bilateral frontal poles. Storage and updating of information within WM can be examined using the n-back, a task where the participant is presented with a series of discrete stimuli and asked to match the current stimulus to one shown n items prior (14). The n-back is an extremely versatile task base, able to utilize multiple stimuli types yet still consistently activate fronto-parieto-cerebellum regions (15, 16) and consistently shows larger recruitment of these areas with increased load (17).

In depression, the n-back task has been used during fMRI scans to examine WM function. Typically, those with depression show larger activation in frontal areas (18–20) which suggest the need to recruit additional neuronal resources to compensate for deficiencies in update. Behavioral evidence for this however has been mixed, fMRI studies have tended to find no significant differences in reaction times (RT) or accuracy (18–23) while purely behavioral studies utilizing identical tasks (24, 25) have found those with depression perform significantly worse than controls.

The processing of stimuli containing emotionally salient information is another area in which differences have been suggested between those with depression compared to healthy controls (4). One commonly reported change from behavioral studies is a bias toward negative stimuli. For example, studies examining processing of words have found those with major depressive disorder (MDD) showed longer RT when attempting to process words that contain negative or depressive related words (26). When processing facial affect, those with depression show a similar bias toward faces displaying negative emotions, and could more quickly identify faces showing emotions such as sadness and disgust, but seem unable to effectively break off attention from the stimuli (27–29). Depressed participants are also more likely to rate neutral faces as sad and happy faces more negatively than controls (30).

Functional studies of emotional processing in depression using a variety of tasks and have found differences in BOLD activity in the putamen, amygdalae / parahippocampal area, insula, subgenual, and anterior cingulate and the ventromedial PFC (VMPF) (31–37). One study examining implicit processing of emotional expression in depression (38) found differential patterns of activity in processing sad and happy faces compared to controls. Those with depression showed increased activity in the right fusiform gyrus, left putamen, and left parahippocampal/amgydalae areas when presented with sad faces and decreased activity in bilateral fusiform and right putamen to happy emotional expressions.

Examining the interactions between negative affect and WM on cognitive performance, Joormann and Gotlib (39) found those with depression showed longer decision times when presented with negative non-target words using a modified version of the Sternberg test. Levens and Gotlib (40), using an emotional variant of the n-back task found depressed participants displayed faster RT when viewing sad faces but also significantly longer to respond to any faces following the presentation of sad faces. This suggests those with depression have attentional bias toward sad displays of facial affect which facilitates more efficient behavioral response. Additionally, this may indicate that when attention has been captured by negative information, WM processes may have more difficulty clearing the negative information thus producing less effective updating to incorporate new information. Thus, recruitment of additional neural resources within frontal regions similar to load effects could be required order to maintain satisfactory performance (18), however no studies have examined this directly.

Therefore, we created an emotional variant of the n-back task for use in fMRI to examine potential neural correlates of WM interruption in facial affective processing in depression. We hypothesized the task would elicit group differences in neural response to facial affect, when comparing the depressed group with controls. Furthermore, we hypothesized the depressed group would show faster RT to emotionally negative (sad) stimuli and increased response in frontal and limbic regions compared to the control group. We also predicted the control group would show similar behavioral and BOLD response, but to emotionally positive (happy) faces.

## Materials and Methods

This pilot study was approved by the Alfred Hospital and Swinburne University of Technology ethics committees in accordance with the declaration of Helsinki.

### Participants and Prescan Procedure

Participants were recruited from the public via social media, advertising in outpatient clinics, patient support groups and referral by mental health professionals. Inclusion in the study required participants to be between the ages of 18–65 with no history of/or suspected neurological disorder, drug or alcohol abuse. Participants were also required to have normal or corrected to normal vision to see the stimuli while in the scanner. In addition, for inclusion in the depression group, participants were required to have their diagnosis confirmed by their treating mental health care professional (psychologist/psychiatrist/GP) and be experiencing a depressed episode. Depression participants were excluded if they had a co-morbid diagnosis, such as bipolar disorder or schizophrenia; anxiety disorders were acceptable, however no participant reported a current diagnosis.

Inclusion was also based on a cut-off score from the depression sub scale of the Depression, Anxiety, Stress Scale 21 (DASS-21) a 21-item questionnaire that measures symptoms associated with depression, anxiety and stress. The DASS-21 has been shown to have good psychometric properties and is strongly correlated with other depression symptoms scales such as the Beck Depression Inventory (41). The DASS-21 is clinically sensitive to severity of state depressive symptoms (42) and their changes over time over time (43).

Control participants were excluded from the study if they scored above a “mild” rating (12+) on the depression sub scale, while those with depression were excluded if they scored below a “moderate” rating (<14). General fMRI safety exclusion criteria were also enforced.

On the day of the scan, participants were asked to fill in a questionnaire package containing demographics questions and a second sitting of the DASS-21. Data from participants whose scores were not within the cut-offs for their respective groups were not included in the study. Participants were then given time outside the scanner to practice the task until they achieved an accuracy of 65% or greater. Those participants requiring corrected vision were fitted with MR compatible prescriptive goggles (mediglasses; http://www.crsltd.com) and tested using a standard Snellen eye chart to confirm 6/6 vision.

Initially 17 controls and 15 depressed participants were recruited, with none reporting inability to perceive colors. Data from 3 controls (2 female) and 2 depressed participants (both male) were not included due to not satisfying the cut-off criteria. The remaining 14 controls and 13 depression participant's demographic information is shown in Table 1.

**Table 1:** Participant demographic information.

	**Control**	**Depressed**
Age in years M (SD)	29.79 (9.62)	30.62 (9.99)
Sex M/F	6/8	3/10
Medication status unmedicated/medicated	N/A	4/9
Length of MDD diagnosis in years M (SD)	N/A	9.48 (7.22)
Number of depressive episodes M (SD)	N/A	9.23 (13.22)
DASS Depression M (SD)	1.43 (1.45)	28.62 (6.99)
DASS Anxiety M (SD)	1.43 (1.22)	21.38 (10.60)
DASS Stress M (SD)	5.71 (4.95)	27.08 (9.54)

Control participants scored within the normal range depression, anxiety, and stress symptoms, while the depressed group were in the severe range for all three (41). Of the nine participants on antidepressant medications, four were on selective serotonin reuptake inhibitors, four on serotonin-norepinephrine reuptake inhibitors and one on anti-psychotics. No participants reported changes within the last 6 months to the type and amount of medication they were taking and none reported a history of electroconvulsive therapy.

#### 3-Back Task

We designed a 3-back version of the well validated n-back task (11) that incorporated two types of stimuli, facial affect and basic visual patterns. The basic visual stimuli were intended to act as a WM baseline, to leave only regions relating to face and emotional processing. Previous studies (44, 45) have found robust emotional effects using a similar contrast. A 3-back design was used due to suggestions by Harvey et al. (18) that hyper frontal responses in depression can only be elicited by tasks requiring large cognitive effort.

#### Task Design and Presentation

Participants were asked to determine whether the sex of the faces or the color of the patterns on the currently presented image was the same or different as the one presented three images ago and ignore the emotion and identity of faces or pattern design. Responses for same or different were obtained through a button box. The first three trials were marked as different with the following stimuli requiring a response of same or different.

The blocked design n-back task consisted of 32 blocks recorded in two runs (16 per run). Each block was 16.2 s in length and consisted of 9 trials, with each trial made up of a 2 s fixation cross and 1.6 s presentation of the stimuli, either faces (male and female faces displaying one of three emotional expressions) or patterns (basic stimuli of patterns and colors with one of three designs). Blocks alternated between faces and patterns conditions, with one emotion (happy, sad, neutral) or pattern design type (horizontal, vertical, checked) presented per block. Participants were asked to determine whether the sex of the faces or the color of the patterns on the currently presented image was the same or different as the one presented three images ago and ignore the emotion and identity of faces or pattern design. Responses for same or different were obtained through a button box. The first three trials were marked as different with the following stimuli requiring a response of same or different. Six blocks of happy emotional expressions and horizontal orientated gratings were recorded over the two sessions (three per session), while the sad, neutral, vertical, and checked blocks were presented five times over the two runs (either two or three presentations per run). Each session was 8.37 min in length with total scan time 16.74 min.

**Figure 1:**
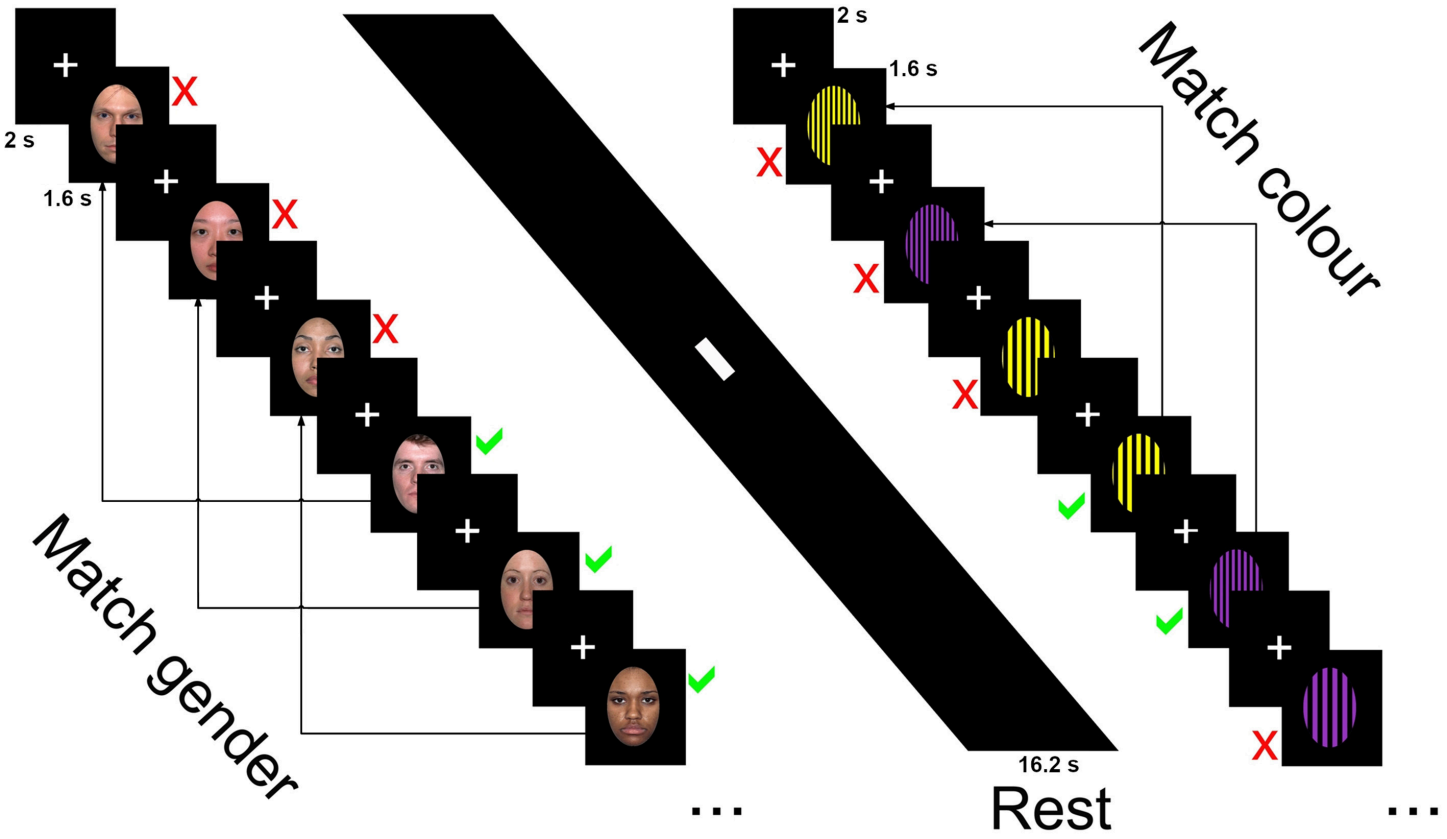
Stimuli presentation sequence for the 3-back task showing neutral faces, rest, and horizontal grating conditions.

The faces condition contained 33 happy, sad and neutral faces (16 female) from the NimStim collection (http://www.macbrain.org/resources.htm), a freely available collection of emotional face stimuli with good internal validity and reliability (46). Faces were closed mouth and contained within a black border. Patterned stimuli were made using Adobe Photoshop CS2 (http://www.photoshop.com) and consisted of three black geometric designs on a colored background—horizontal stripes, vertical stripes, or a checked pattern. Backgrounds consisted of 2 colors, purple or yellow with patterns made of widths ranging from 5 mm line widths to 12 in 1 mm increments (7 increments per pattern, 21 in total). Stimuli were presented using E-Prime version 2 (www.pst.com) and presented on a 24-inch (60.96 cm) Cambridge research systems BOLDscreen fMRI (http://www.crsltd.com). Participants viewed the images through a mirror mounted on the head coil, roughly 10 cm from the eyes and 100 cm from the monitor. Figure 1 displays the stimuli presentation sequence.

#### Behavioral Analysis

Behavioral data and correlations between fMRI and clinical data were analyzed using the python 2.7 packages scipy and pyvttbl (https://github.com/rogerlew/pyvttbl). Custom permutation tests (47) based on mixed ANOVAs (48) two tailed Wilcoxon signed-rank *post-hoc* tests (49) and two tailed Spearman correlation coefficients (modified from the method proposed by (50) were used for null hypothesis testing. The single step maxT method (51) was used for correction of multiple comparisons in *post-hoc* and correlation analyses. For each test, 10,000 re-shuffles were used. Scripts are available from http://github.com/petergoodin/montecarlo_funcs or on request.

#### fMRI Acquisition

Data were collected with a Siemens Tim Trio 3T using a standard 32 channel head coil. A high resolution MPRAGE scan (208 slices, TR = 1,800 ms, TE = 2.79 ms, inversion time = 900 ms, slice thickness = 0.7, in plane resolution of 0.7 mm3, FOV 210 mm, flip angle 9°, 320 frequency/phase encoding steps) was collected for registration to the functional data and normalization to a standardized space. Whole brain fMRI data were collected using an echo planar imaging (EPI) sequence, AC/PC aligned axial acquisition (TR = 3,000 ms, TE = 30 ms) with 168 volumes containing 51 slices (slice thickness = 2.5 mm with a 0.5 mm gap between slices, in plane resolution of 3 mm3, 74 × 74 matrix, flip angle = 90°, 74 frequency/phase encoding steps, bandwidth = 1648 Hz per pixel, GRAPPA factor 2).

#### fMRI Analysis

fMRI data preprocessing and modeling was carried out using FEAT and MELODIC, part of the FSL 5.0.9 package (www.fmrib.ox.ac.uk/fsl). Functional data were unwarped using fieldmaps acquired prior to each run (TE = 30, blip direction = negative) and registered to a 2 mm MNI space template brain using FLIRT. Preprocessing consisted of motion correction using MCFLIRT, slice timing correction (52) using Fourier-space time-series phase-shifting, application of a 6 mm full width half maximum (FWHM) smoothing kernel and intensity normalization across volumes. Optimal high pass filtering was calculated automatically in FEAT using the first level design matrix and used Gaussian-weighted least-squares straight line fitting with sigma = 45 s (0.022 Hz).

Independent Component Analysis (ICA) using MELODIC was used to decompose the data to manually remove artifacts. Components were deemed to be artifactual if: time series that did not resemble the known blocked onset/offset times of the stimuli; power spectral densities were clustered toward higher frequencies, extremely low frequencies or had a relatively uniform distribution. Components were also deemed artifactual if their spatial characteristics resembled known artifact types (e.g., radio frequency artifact producing a random speckled pattern, motion artifact showing activation around the edge of the brain or large extremely smooth regions with no tissue boundaries, cardiac/vasculature artifact showing BOLD response localized in vessels such as the carotid and middle cerebral arteries, and responses within white matter and cerebrospinal fluid filled regions including ventricles).

First level regressors were convolved with a double gamma function with inclusion of temporal derivatives. Each emotion condition (happy, sad, and neutral) were contrasted against the mean response of the patterned conditions. A second level fixed effects analysis was used to combine the two runs and the resulting contrast of parameter estimates (COPE). Higher level, within and between-group contrasts were examined using Randomize (53). Pooled one sample *t*-test permutation analyses were performed for each emotional condition to show location of activation using threshold-free cluster enhancement (TFCE) (54) and 10,000 re-shuffles. Two sample contrasts (control minus depressed) were also performed for each emotional condition to examine for differences of emotional processing on BOLD response during the n-back task. The Harvard Oxford atlas (55) was used for neuroanatomical labeling. Group level difference significant cluster mean estimates were extracted across individuals for correlation analyses with behavioral data (56).

For all analyses, a maxT corrected alpha of 0.05 or less was considered the threshold to signify a result being of sufficiently low probability as occurring under the null distribution to warrant consideration as a potentially legitimate effect (significant).

## Results

### Behavioral Data

A two sided Fisher's exact test showed no significant imbalance of sex between the control and depressed group (Odds ratio = 2.5, *p* = 0.419). Permutation analysis showed no significant difference in age (t-obs = 0.21, *p* = 0.816) and revealed the expected increased symptom scores for depression, anxiety and stress scales (all t-obs > 7.01, all *p* < 0.001) for the depressed group.

Table 2 outlines the means (M) and standard deviations (SD) for accuracy and RT, between the control and depression groups across the six conditions and between condition significant differences.

**Table 2:** Mean (SD) for accuracy (%ACC) and reaction time (RT) of control and Depressed participants across happy, sad, neutral faces and vertical, horizontal, checked pattern conditions.

	**%ACC M (SD)**		**RT M (SD)**	
	**Control**	**Depressed**	**Control**	**Depressed**
Happy	0.71 (0.19)	0.70 (0.15)	794.15 (129.47)	820.60 (81.81)
Sad	0.74 (0.19)	0.73 (0.10)	778.21 (133.11)	811.87 (108.71)
Neutral	0.79 (0.17)	0.74 (0.11)	763.62 (147.57)	807.26 (104.58)
Vertical	0.78 (0.19)	0.75 (0.13)	684.47 (139.98)	736.87 (98.15)
Horizontal	0.75 (0.18)	0.77 (0.15)	667.86 (153.32)	672.34 (84.55)
Checked	0.80 (0.19)	0.79 (0.12)	680.33 (136.86)	718.47 (89.61)

**Figure 2:**
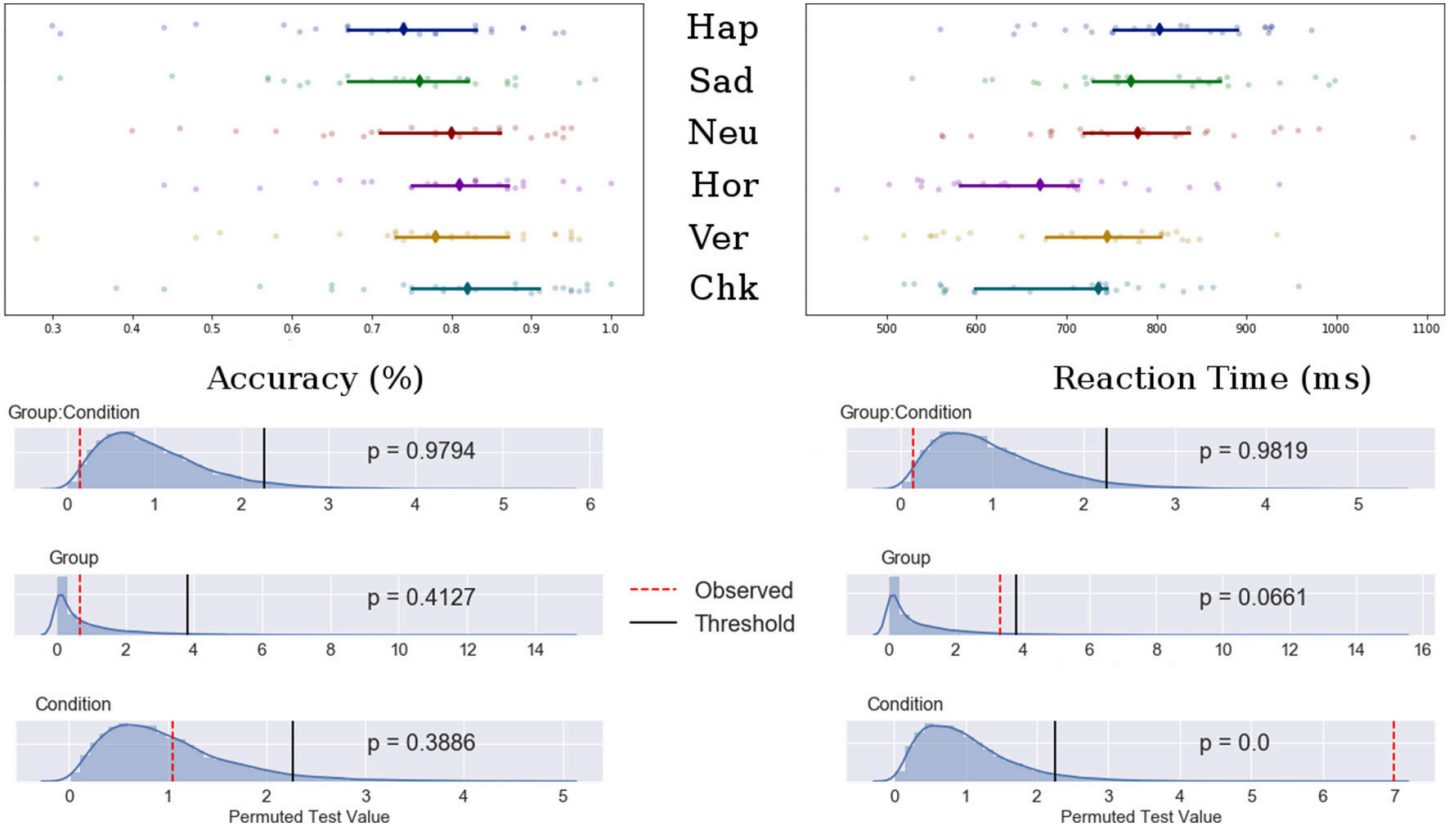
**Top**: Median value for accuracy **(Left)** and reaction time **(Right)** for happy, sad, neutral, horizontal, vertical, and checked conditions. Bars indicate 95% confidence interval bootstrapped 10,000 times, points represent individual participant data. **Bottom**: Permuted null distribution with observed (red) and threshold (black) values marked as vertical bars.

Potential main effects and interaction differences in n-back accuracy and reaction time were tested using separate permutation tests using 3 (emotional condition) × 2 (Group) mixed ANOVAs. Results showed significant main effects of condition for both accuracy (F-obs = 9.00, *p* < 0.001) and reaction time (F-obs = 27.17, *p* < 0.001) but not for group (F-obs < 1.00, *p* > 0.40). There was no significant group interaction for either accuracy or reaction time (F-obs < 1.30, *p* > 0.27). The permutation results are shown in Figure 2.

*Post-hoc* analyses showed decreased accuracy for the happy condition compared to all patterned and neutral conditions (t-obs < −3.40, *p* < 0.01), sad and neutral to checked pattern conditions (t-obs < −2.81, *p* < 0.05) and sad to neutral conditions (t-obs = −2.59, *p* = 0.48). Accuracy was decreased for the vertical compared to the checked pattern conditions (t-obs = −2.64, *p* = 0.043). RT were significantly increased for the emotional conditions compared to patterned conditions (t-obs > 3.01, *p* < 0.001). No differences were found in reaction times between emotions. The vertical condition stimuli were reacted to slower compared to horizontal conditions stimuli (t-obs = 3.01, *p* = 0.041).

### Task fMRI Data

**Figure 3:**
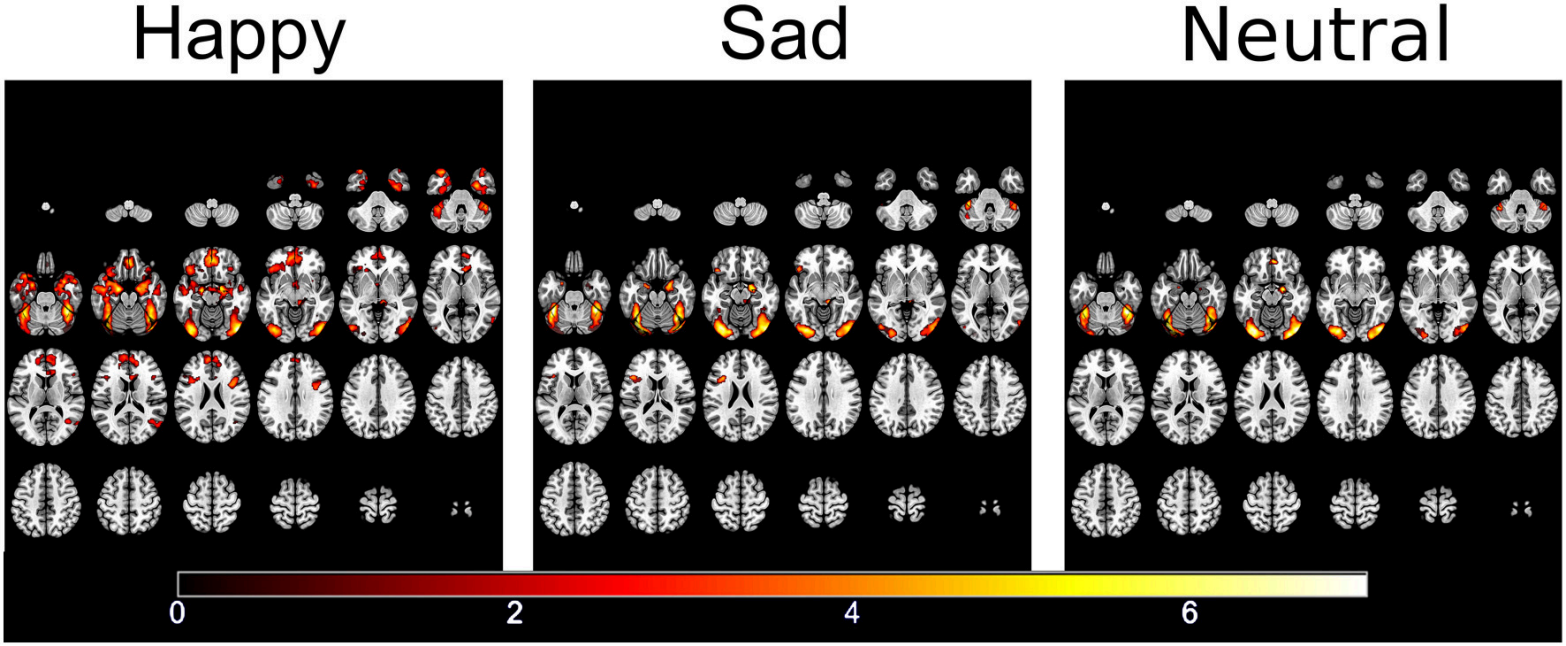
Significantly activated areas for happy, sad, and neutral faces compared to the patterned baseline.

fMRI responses to each emotion pooled across the two groups showed activation within face and emotional processing regions including the fusiform gyrus, temporal poles, amgydalae and inferior frontal gyrus (57) (see Figure 3).

**Figure 4:**
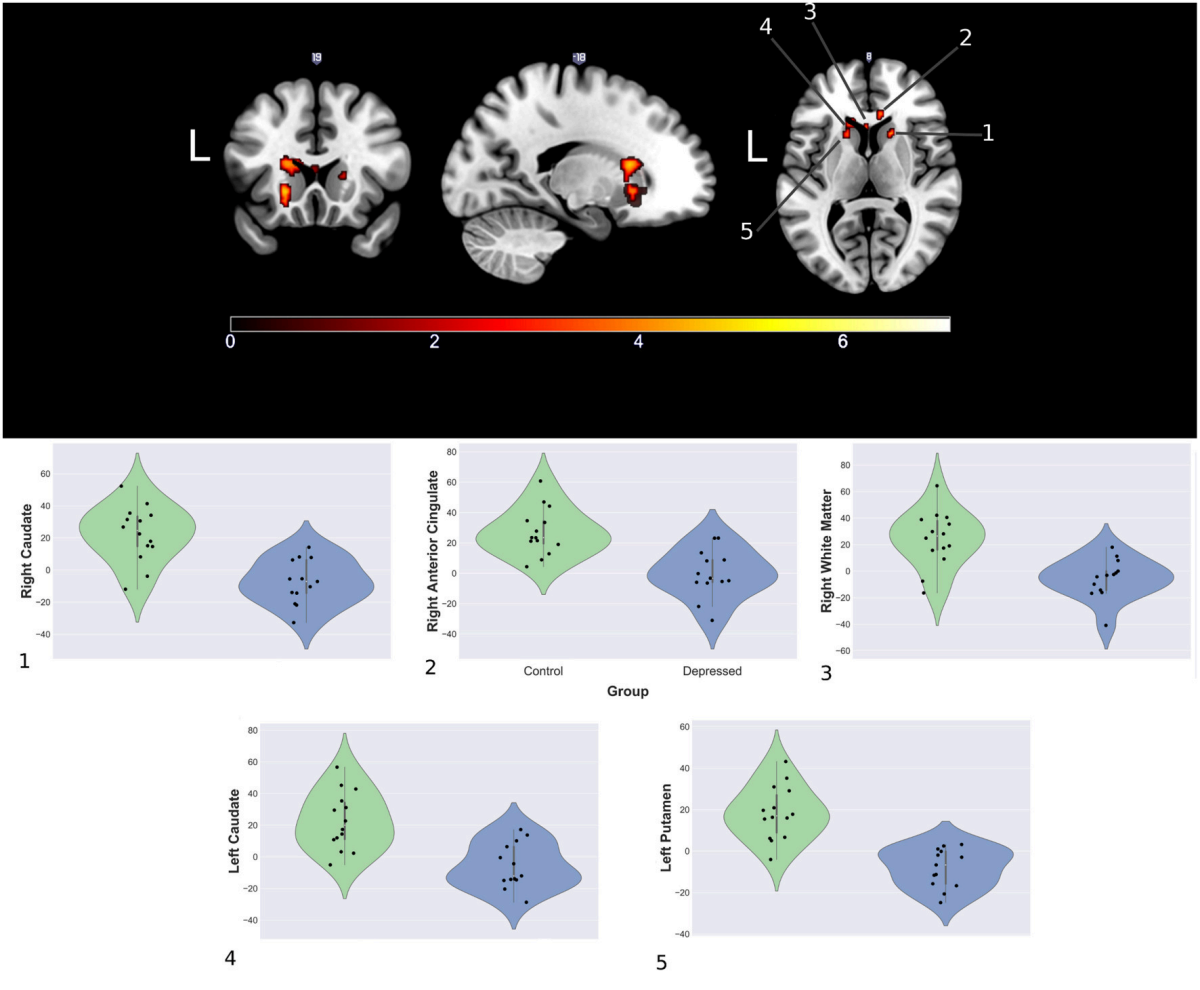
Significantly increased areas of activation in the control group compared to depression during presentation of happy faces **(Top)** and mean cluster estimates **(Bottom)**.

Between group analyses showed significantly increased activation for control compared to depressed participants in response to happy faces within the striatum (left and right caudate, left putamen), genu of the corpus collosum and the right rostral anterior cingulate (rACC). Extracted contrast estimates showed control participants had activation greater than the patterns baseline, while depressed participants generally showed responses similar to or less than baseline. Areas showing significant differences between the groups and individual contrast estimate values are shown in Figure 4. No significant differences were found between groups for sad and neutral conditions (see Supplemental Material for cluster sizes, peak locations, and peak value).

### Clinical—fMRI Correlations

**Figure 5:**
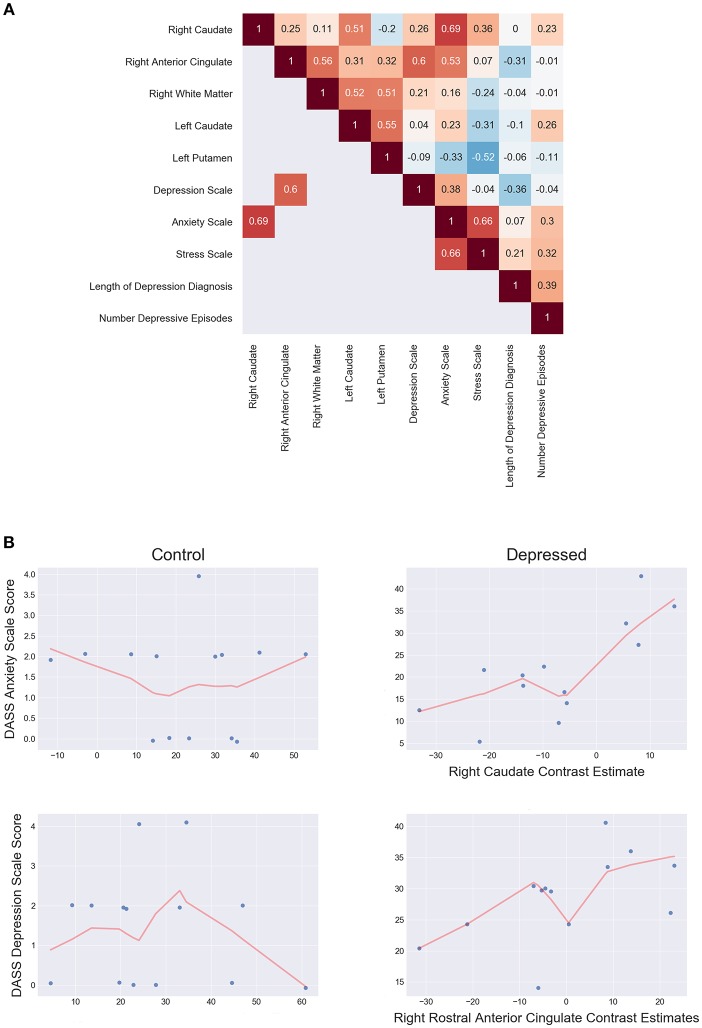
**(A)** Depressed correlation matrix of Spearman's ρ shows unthresholded correlations in the top triangle and thresholded (*p* < 0.05 two tailed) correlations on the bottom. **(B)** Scatterplots of controls (left) and depressed (right) for DASS anxiety scale score and right caudate estimates (top) and DASS Depression scale score and right rostral anterior cingulate estimates (bottom). LOESS line (red; fraction = 0.5) indicates the direction of the monotonic relationship.

*Post-hoc* spearman correlation analyses on mean cluster estimates and clinical scores found two significant, moderately positive monotonic correlations for the depressed group. Increased right rACC response were associated with increased DASS-21 depression scale score (ρ = 0.60, *p* = 0.036), while increased right caudate response correlated with increased with DASS-21 anxiety scale score (ρ = 0.69, *p* = 0.013). There were no significant clinical and fMRI correlations for the control group. Further analysis showed the depressed group had significantly increased correlations between the rACC and DASS-21 depression score (Δ ρ = 0.57, *p* = 0.009) and the right caudate with anxiety (Δ ρ = 0.755, *p* ≤ 0.001) when compared to controls. Figure 5 shows the correlation matrix for the depressed group and scatterplots between the rACC and DASS depression score and the right caudate with anxiety (see Supplemental Material for the control group's correlations).

## Discussion

Our novel n-back task was able to elicit group level BOLD differences in facial affectual processing when comparing depressed and control groups, which was as predicted. To our knowledge, this is the first study that has examined BOLD response to emotional faces during working memory update.

Contrary to expectations, the depressed group did not show the predicted decrease in reaction time (RT) or increased BOLD response to sad faces in frontal regions when compared to controls. Controls also did not show faster RT and increased frontal BOLD to happy faces. Depressed participants displayed decreased BOLD activation in several regions of the striatum and the rACC in response to happy faces when engaged in the n-back task. Extracted contrast estimates showed the average depressed group response to happy was similar to the baseline patterns condition. Additionally, depressed participants also showed significant, moderate, positive monotonic correlations between DASS-21 depression symptom score with the rACC, and the DASS-21 anxiety symptom score with the right caudate.

We were unable to detect the hypothesized differences in RT between happy and sad faces. Slower RT to negative stimuli in those with depression have been previously reported in behavioral studies (4, 40), however in this study we found no significant difference between the control and depressed group. This consistent with other emotional expression n-back studies conducted using fMRI (18, 20, 21). We were also unable to detect changes within the frontal and limbic systems as hypothesized. Altered frontal and limbic activity, especially the ventromedial prefrontal cortex (VMPFC) and amygdalae reactivity are a commonly reported functional association with depression (58–60), however we did not detect any significant differences between the two groups contrary to our hypothesis.

This study found decreased response in the striatum in response to happy faces in the depressed group. Part of the dopamine innervated basal ganglia, the striatum has is associated addiction, motivation and inhibitory behaviors (61–63). The dorsal striatum, which includes the caudate and putamen is considered to link frontal “top-down” and more posterior “bottom-up” driven systems involved in attention, memory, social and emotional processing, integrating information, and functioning in a goal directed, motivated capacity (64). Fiber tracking studies have found projections from the putamen connect to the primary motor cortex and supplementary motor area (65, 66) while the caudate has dense connections with largely frontal regions, including the prefrontal cortex (67), an area strongly associated with WM (11).

Like the dorsal striatum, the anterior cingulate has been associated with a substantial amount of stimuli responsivity and behavioral outcomes (68, 69) however focused studies and parcellation of the region (70) has allowed for some delineation of functioning within subregions (71, 72). Projections from the rACC connect largely with the prefrontal cortex in addition to tracts between the rACC and limbic regions, including the amygdala (73). Functional connectivity has shown anti-correlations between the rACC with motor and dorsolateral prefrontal regions, while medial structures such as the limbic and orbitofrontal cortex (OFC) show positive correlations (74). It has been suggested that the region may be involved with emotional awareness or orientating attentional resources to the processing of emotional stimuli (75), of which preference in healthy individuals may be given to happy faces (76, 77).

Our results showed the depressed group had lower fMRI response to happy faces when compared to controls. Additionally, the decreased response was not greater than the baseline, which may indicate that as a group, the depressed cohort failed to differentiate between visual displays of positive emotion and visual information of no emotional salience. Depression is associated with alterations in emotional processing, which can include decreased recognition of positive emotion, such as happy faces (35, 40, 78). Several fMRI studies have shown changes in functional connectivity between the dorsal striatum and other regions of the brain affect emotional processing in depression (79–81). For example, Yang et al. (82) have shown decreased activation in the extended regions including the striatum only when viewing positive emotional stimuli. Furthermore, they found this decreased activity and resting state functional connectivity correlated with clinical scores.

Additionally, several functional imaging meta-analyses (58–60, 83) have found the rACC, caudate and putamen display decreased BOLD signal to positive emotional stimuli in depressed individuals. Murrough et al. (84) found depressed participants also displayed decreased response to happy faces compared to healthy controls in the right caudate. When administered a single dose of ketamine, activation in the caudate increased significantly compared to baseline levels. This suggests the striatum may be involved in emotional blunting to positive affect. The Positive Attenuation Hypothesis (PAH), posits those with depression have decreased response to positive emotional stimuli compared to healthy controls, stemming from depressed individual's tendency to exhibit low positive mood (85, 86). Decreased response to positive emotional stimuli may arise from motivational deficits (87). Given the dorsal striatum's association with reward processing, attention and motivation and its role in emotional processing the results of our study suggest those with depression may have decreased reward response and motivation to engage with positive stimuli during working memory update, potentially due to inability to differentiate between emotional conditions. Whether this is a prodromal response or a trait caused by depressive episode onset is unknown.

Positive monotonic associations were observed between depression symptom score with the rACC and anxiety symptom score with the right caudate. Several studies have found functional connectivity to the striatum to largely frontal regions is associated with clinical variables (82, 88, 89) however this is the first study to show region specific correlations with clinical variables during a working memory task. Of note, these associations were significantly larger for the depressed group compared to the control group, who showed weak associations between these regions and clinical scores. Given the positive nature of the relationship, this suggests as activation in these regions for depressed participants increases, so too does the clinical symptom scores. However, the control group showed increased activation in these regions compared to the depressed group but did not show any association. These results may suggest there may be altered functionality in the striatum of those with depression that extends beyond simple differences in activation. It may be beneficial for future studies to validate this dissociation with larger sample.

Some limitations to the study need to be acknowledged. This study had a small sample size with 13 depressed and 14 control participants included in the final analysis. This typically leads to overfitting to the observed data and inability to generalize the results to the wider populations of interest. For these reasons we utilized permutation testing which allows for inference regarding the samples directly with good control of type 1 error and with confidence of the reported *p*-value directly linked to the number of reshuffles (53). While our results may not be able to be directly applied on the depressed population, they may still be used as to inform further study and be validated through replication, like other studies that utilize the null hypothesis testing *framework* (90). We did not detect the predicted decreased reaction time and increased frontal activation for depressed participants to sad faces. This may be due to the small sample size of this study which was insufficient to detect the effect size of interest and stratify depression behavioral subtypes. McIntyre et al. (91) has suggested overt WM deficits in behavioral outcomes such as accuracy and RT in depression have small to medium effects sizes. Furthermore, they suggested that distinct deficits in working memory occur only within 20–30% of those with the disorder. The small participant numbers found in many neuroimaging studies, including this one, would be unable to differentiate between such small effect sizes. Additionally, despite showing severe levels of depressive symptoms as measured by the DASS21, the depressed group were all recruited from the general public and were able to function within wider society, taking part in normal activities such as attending work, school, and raising families. These participants were able to organize their time, attend and engage in the testing session. This suggests the depressed participants recruited for this study may have developed successful methods to compensate for their disorder and, in turn, this may have influenced the behavioral results.

We were also unable to detect difference in amygdalae response between groups. This may have been due our assumption that the presentation of faces using a blocked design would elicit a consistent response in the amygdalae over time. However, amygdalae response to constant presentation of affectual stimuli may habituate (92). The constant presentation of faces with the same emotional expression during a block may have attenuated amygdalae response. Further, the decreased amygdalae response may have also been associated with lack of detected difference in the VMPFC. Roy et al. (93) suggested a role for the VMPFC is inhibition of subcortical / limbic responses. As the response of the amygdalae attenuated, it is possible the inhibitory response of the VMPFC was also decreased. Revising the design to be event related may help to identify if this was the cause.

In this study, 4 of the 13 depressed participants (~30%) were on pharmacological treatment for their condition. This may have been another reason for the lack of difference between the depressed and control group. Medication effects have also been reported to alter response in the caudate nucleus and anterior cingulate during a verbal n-back task (23), with medication reported to increase the BOLD response in these areas. In our study we found the depressed group displayed significantly decreased response to happy faces compared to controls in these regions, so assuming a similar response had medicated participants not been included, we may have observed an even more exaggerated decrease in BOLD response.

In conclusion, this study aimed to examine neural correlates of WM update when processing displays of facial affect using a novel emotional n-back variation. We found the depressed group showed a failure to activate to positive emotional stimuli compared to healthy controls in the dorsal striatum and rACC. Additionally, the right caudate activity associated with increased depressive symptom score. This suggests the task we developed to probe WM update in depression to facial affect can elicit an emotional response between control and depressed participants while performing the n-back task. Furthermore, our preliminary results suggest those with depression exhibit altered functionality of the dopaminergic dorsal striatum which contributes to depressed mood through attenuation of positive emotional stimuli during WM update. Further examination into the dorsal striatum's relationship with emotional processing in working memory may contribute to our understanding of decreased responsiveness to positive information in depression.

## Author Contributions

PG, SR, and JC conceived and designed the study. PG oversaw data collection and integrity, wrote the permutation methods to analyse the behavioral data, analyzed behavioral, and fMRI data, and wrote the manuscript drafts. SR and JC provided feedback on the manuscript drafts. MH provided domain knowledge and feedback on the manuscript drafts. GL provided extensive domain knowledge and feedback on the manuscript drafts.

### Conflict of Interest Statement

The authors declare that the research was conducted in the absence of any commercial or financial relationships that could be construed as a potential conflict of interest.
