# Supplementary material for: Decreased Response to Positive Facial Affect in a Depressed Cohort in the Dorsal Striatum During a Working Memory Task—A Preliminary fMRI Study
Source: Front Psychiatry. 2019 Mar 5;10:60. doi: 10.3389/fpsyt.2019.00060 (PMC6411826; doi:10.3389/fpsyt.2019.00060)

**Supplemental material:**

**Pooled happy co-ordinates**

Peak activations during the happy condition.

| **Region** | **Cluster size** | **x** | **y** | **z** | **t value** |
| --- | --- | --- | --- | --- | --- |
| R Lat Occipital | 11424 | 48 | - 80 | - 14 | 3.6 |
| L Med Frontal | 2346 | - 4 | 44 | - 18 | 3.42 |
| R Precentral | 315 | 42 | 8 | 26 | 3.78 |
| R Wm | 153 | 8 | 28 | 4 | 3.46 |
| L Inf Frontal | 142 | - 42 | 20 | 18 | 3.35 |
| R Ant Cingulate | 62 | 6 | 20 | 20 | 3.29 |
|  |  |  |  |  |  |
| **Control > MDD** |  |  |  |  |  |
| L Caudate | 144 | -18 | 16 | 14 | 4.99 |
| L Putamen | 100 | -20 | 18 | -4 | 5.10 |
| R Caudate | 25 | 16 | 16 | 10 | 4.99 |
| R Wm | 10 | -2 | 20 | 10 | 4.36 |
| R Ant Cingulate | 6 | 8 | 32 | 8 | 4.26 |
|  |  |  |  |  |  |
| **MDD > Control** |  |  |  |  |  |
| Null |  |  |  |  |  |
|  |  |  |  |  |  |

L = Left, R = Right.

Ant = Anterior, Post = Posterior, Med = Medial, Lat = Lateral, Sup = Superior, Inf = Inferior.

Wm = White matter.

**Pooled sad co-ordinates**

| **Region** | **Cluster size** | **x** | **y** | **z** | **t value** |
| --- | --- | --- | --- | --- | --- |
| R Fusiform | 2846 | 40 | - 50 | - 26 | 9.31 |
| L Fusiform | 2632 | - 40 | - 50 | - 28 | 8.47 |
| R Amygdala | 267 | 20 | - 4 | - 18 | 6.98 |
| L Inf Frontal | 198 | - 32 | 14 | 22 | 5.6 |
| R Parahippocampal | 94 | 12 | - 32 | - 12 | 7.22 |
| L Frontal Orbital | 77 | - 44 | 28 | - 12 | 5.92 |
| L Amygdala | 40 | - 22 | - 2 | - 22 | 5.78 |
|  |  |  |  |  |  |
| **Control > MDD** |  |  |  |  |  |
| Null |  |  |  |  |  |
|  |  |  |  |  |  |
| **MDD > Control** |  |  |  |  |  |
| Null |  |  |  |  |  |

L = Left, R = Right.

Ant = Anterior, Post = Posterior, Med = Medial, Lat = Lateral, Sup = Superior, Inf = Inferior.

Wm = White matter.

**Pooled neutral co-ordinates**

| **Region** | **Cluster size** | **x** | **y** | **z** | **t value** |
| --- | --- | --- | --- | --- | --- |
| R Lat Occipital | 2288 | 48 | - 80 | - 12 | 10.7 |
| L Fusiform | 2177 | - 32 | - 84 | - 18 | 8.83 |
| R Amygdala | 92 | 20 | - 8 | - 14 | 6.96 |
| R Med Frontal | 53 | 6 | 42 | - 16 | 6.69 |
| L Amygdala | 2 | - 20 | - 4 | - 22 | 6.25 |
|  |  |  |  |  |  |
| **Control > MDD** |  |  |  |  |  |
| Null |  |  |  |  |  |
|  |  |  |  |  |  |
| **MDD > Control** |  |  |  |  |  |
| Null |  |  |  |  |  |

L = Left, R = Right.

Ant = Anterior, Post = Posterior, Med = Medial, Lat = Lateral, Sup = Superior, Inf = Inferior.

Wm = White matter.

**Correlation matrix controls**


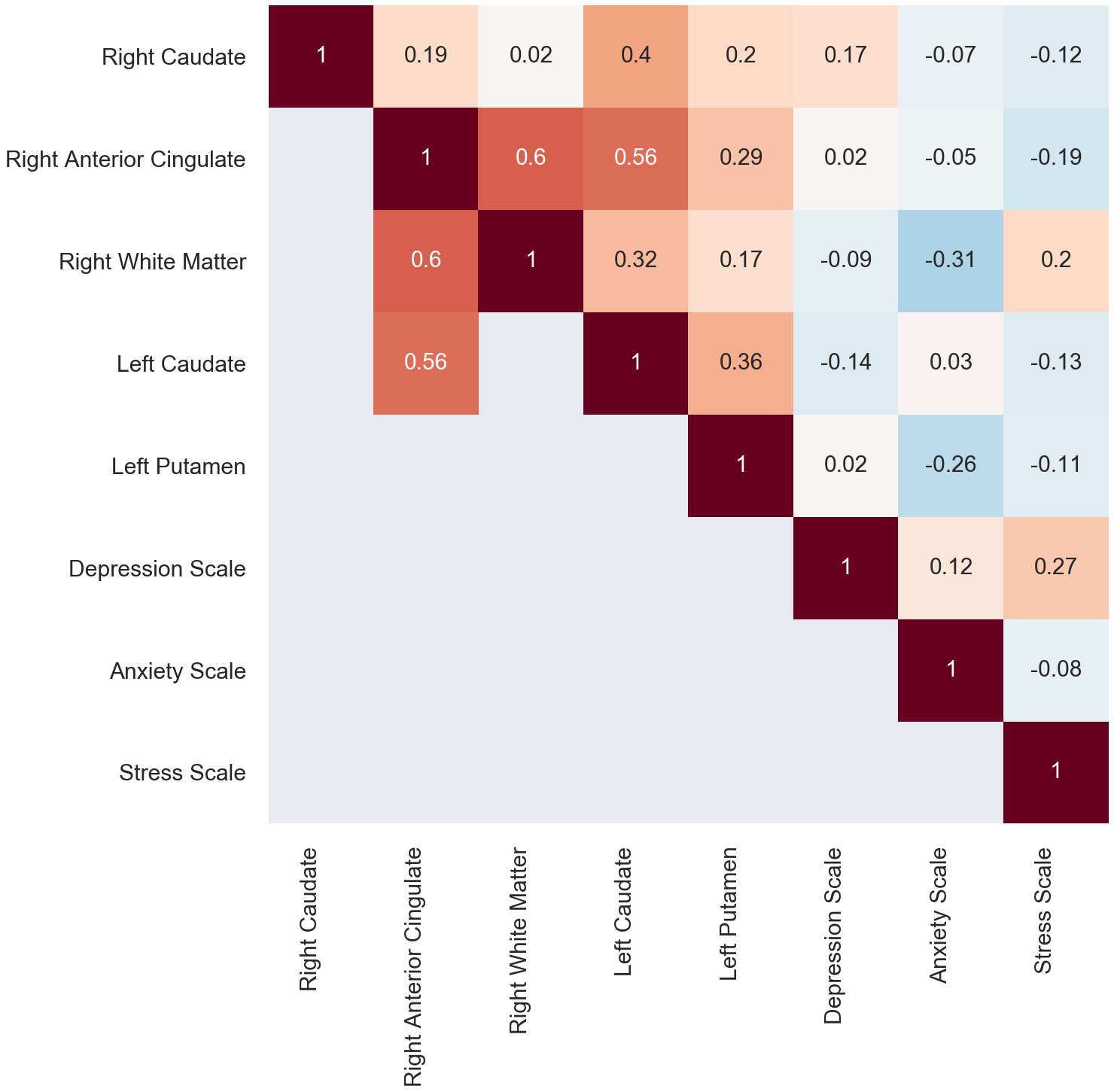

Supplement: Supplementary file 1 [file Data_Sheet_1.docx]
